# Xanthine Oxidoreductase: A Double-Edged Sword in Neurological Diseases

**DOI:** 10.3390/antiox14040483

**Published:** 2025-04-17

**Authors:** Massimo Bortolotti, Letizia Polito, Maria Giulia Battelli, Andrea Bolognesi

**Affiliations:** Department of Medical and Surgical Sciences—DIMEC, Alma Mater Studiorum, University of Bologna, Via San Giacomo 14, 40126 Bologna, Italy; massimo.bortolotti2@unibo.it (M.B.); mariagiulia.battelli@unibo.it (M.G.B.); andrea.bolognesi@unibo.it (A.B.)

**Keywords:** xanthine oxidoreductase, uric acid, neurodegenerative diseases, stroke, oxidative stress, inosine

## Abstract

Non-communicable neurological disorders are the second leading cause of death, and their burden continues to increase as the world population grows and ages. Oxidative stress and inflammation are crucially implicated in the triggering and progression of multiple sclerosis, Alzheimer’s disease, amyotrophic lateral sclerosis, Huntington’s disease, Parkinson’s disease, and even stroke. In this narrative review, we examine the role of xanthine oxidoreductase (XOR) activities and products in all the above-cited neurological diseases. The redox imbalance responsible for oxidative stress could arise from excess reactive oxygen and nitrogen species resulting from the activities of XOR, as well as from the deficiency of its main product, uric acid (UA), which is the pivotal antioxidant system in the blood. In fact, with the exception of stroke, serum UA levels are inversely related to the onset and progression of these neurological disorders. The inverse correlation observed between the level of uricemia and the presence of neurological diseases suggests a neuroprotective role for UA. Oxidative stress and inflammation are also caused by ischemia and reperfusion, a condition in which XOR action has been recognized as a contributing factor to tissue damage. The findings reported in this review could be useful for addressing clinical decision-making and treatment optimization.

## 1. Introduction

According to the Global Burden of Diseases, Injuries, and Risk Factors Study (GBD), which analysed data collected between 1990 and 2016, neurological disorders were among the main causes of deaths, and they are the leading cause of disability-adjusted life-years, i.e., the sum of years of life lost and years lived with disability. The burden of non-communicable neurological disorders continues to increase as the world population grows and ages because the prevalence of major disabling neurological disorders increases with age [1].

The brain consumes a fifth of the oxygen supplied by the lungs, and this intense metabolic activity results in the generation of reactive oxygen species (ROS). Furthermore, ROS and reactive nitrogen species (RNS) are produced during dopamine oxidation, and white matter is highly sensitive to oxidants, the production of which increases with aging [2]. For these reasons, the antioxidant system is fundamental for central nervous system (CNS) health, and oxidative stress is crucially implicated in the triggering and progression of multiple sclerosis (MS) [3] and other neurodegenerative diseases prevalent in adulthood and old age, such as Alzheimer’s disease (AD) [4], amyotrophic lateral sclerosis (ALS) [5], Huntington’s disease (HD) [6], and Parkinson’s disease (PD) [7]. Oxidative stress is also caused by ischemia and reperfusion, a condition in which the action of xanthine oxidoreductase (XOR) has been recognized as a contributing factor to tissue damage. Therefore, it is worth considering the implication of oxidative stress and antioxidant defences in stroke [8]. In most degenerative diseases and stroke, both neuroinflammation and oxidative stress are involved in the triggering of tissue damage and cell death [9]. The redox imbalance responsible for oxidative stress could derive from an excess of ROS and RNS originating from the activity of XOR [10], as well as from the deficiency of its main product, uric acid (UA), which is the pivotal antioxidant system in blood [11].

In many neurological diseases, including MS, AD, PD, and HD, astrocytes play a relevant role in regulating the inflammatory response, thus becoming potential targets for therapeutic intervention [12].

In this narrative review, we examined the role of XOR activity and products in MS, AD, ALS, HD, PD, and stroke. The bibliography was searched in the PubMed and Google Scholar databases using the keywords “Alzheimer’s disease”, “amyotrophic lateral sclerosis”, “Huntington’s disease”, “multiple sclerosis”, “neurological disorders”, “oxidative stress”, “Parkinson’s disease”, “stroke”, “uric acid”, and “xanthine oxidoreductase” in appropriate combinations.

## 2. Xanthine Oxidoreductase

XOR is produced by human cells as a nicotinamide adenine dinucleotide (NAD^+^)-dependent dehydrogenase (XDH, EC 1.17.1.4), which is converted into an oxidase (XO, EC 1.17.3.2) when it is released into body fluids. This conversion results from the reversible oxidation of a pair of crucial cysteine residues or from the irreversible partial proteolysis of the portion containing these cysteine residues. During the transition from XDH to XO, an intermediate form of XOR is generated that may react with both NAD^+^ and O_2_ [13]. Either XDH, XO, or their intermediate form catalyse the oxidation of hypoxanthine to xanthine and xanthine to UA, which are the terminal and irreversible reactions of purine catabolism in higher primates. This rate-limiting function of XOR precludes the salvage pathway of purine nucleotides.

UA generated by both XDH and XO activities has several physiological functions: (i) it contributes to the maintenance of blood pressure by activating the renin–angiotensin system; (ii) it has a fundamental activity of free radical scavenging in body fluids; (iii) it exerts a pro-inflammatory action by stimulating macrophages as a damage-associated molecular pattern and increasing cyclooxygenase-2 expression; (iv) it influences metabolism by promoting glucose availability and fat accumulation; (v) it stimulates the CNS, in a manner similar to other purines, such as caffeine [11,14,15]. However, hyperuricemia is associated with gout, hypertension, cardiovascular diseases, renal diseases, obesity, diabetes, metabolic syndrome, and tumour lysis syndrome, although only in gout and tumour lysis syndrome has the causal role of UA been ascertained and the urate-lowering therapy proven to be effective [16,17]. UA intracellular accumulation can induce oxidative stress by promoting mitogen-activated protein (MAP) kinases and nicotinamide adenine dinucleotide phosphate (NADPH) oxidase activities and by inhibiting the signalling pathway of the nuclear factor erythroid 2-related factor 2 (NRF2), which induces the expression of antioxidant proteins [18]. In addition, intracellular UA contributes to inflammation and oxidative stress by inducing the expression of cyclooxygenase 2, thromboxane, and chemokines and activating the inflammasome [18]. The concept that both hypouricemia and hyperuricemia can be harmful has been summarized in the term dysuricemia [19].

**Figure 1:**
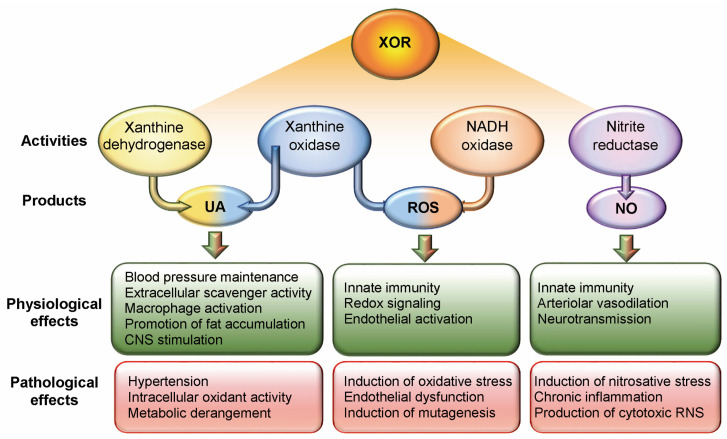
Physiological and pathological effects of xanthine oxidoreductase (XOR) activity and products. Uric acid (UA) generated by both xanthine dehydrogenase and xanthine oxidase activities has several physiological functions. UA excess, on the other hand, is responsible for promoting hypertension, has intracellular oxidative activity, and promotes metabolic alterations such as insulin resistance. Reactive oxygen species (ROS) are produced by both the xanthine oxidase and reduced nicotinamide adenine dinucleotide (NADH) oxidase activities of XOR. Physiological ROS levels have a redox signalling function, favouring the innate immune response by activating both endothelial cells and leukocytes. However, ROS excess causes oxidative stress, promotes endothelial dysfunction, and induces mutagenesis. Nitric oxide (NO) is generated by the nitrite reductase activity of XOR and has several physiological functions. However, excess NO can induce nitrosative stress, promote chronic inflammation, and contribute to the production of cytotoxic reactive nitrogen species (RNS).

Under hypoxic and acidosis conditions, XOR can have reduced nicotinamide adenine dinucleotide (NADH) oxidase and nitrite reductase activity. Both the XO and NADH oxidase activities of XOR produce ROS, hydrogen peroxide, and superoxide anion, which can give rise to the highly cytotoxic hydroxyl radical in the presence of transition metals. Physiological ROS levels have a redox signalling function and favour the innate immune response by activating both endothelial cells and leukocytes and by potentiating their bactericidal action during phagocytosis [11]. XOR-generated ROS activate endothelial cells and leukocytes: endothelial cells increase their permeability and express adhesion molecules, and leukocytes release cytokines that upregulate macrophage XOR expression. However, excess ROS causes oxidative stress, promotes endothelial dysfunction, and induces mutagenesis [20]. The nitrite reductase activity of XOR generates nitric oxide (NO), which in turn can generate cytotoxic RNS, such as peroxynitrite ions. NO has the following functions: (i) it promotes the innate immune response by activating the endothelium; (ii) it regulates local vascular tone by inducing vasodilation; and (iii) it is a retrograde neurotransmitter in synapses [11]. However, excess NO can induce nitrosative stress, promote chronic inflammation, and contribute to the production of cytotoxic RNS [21] (Figure 1).

**Figure 2:**
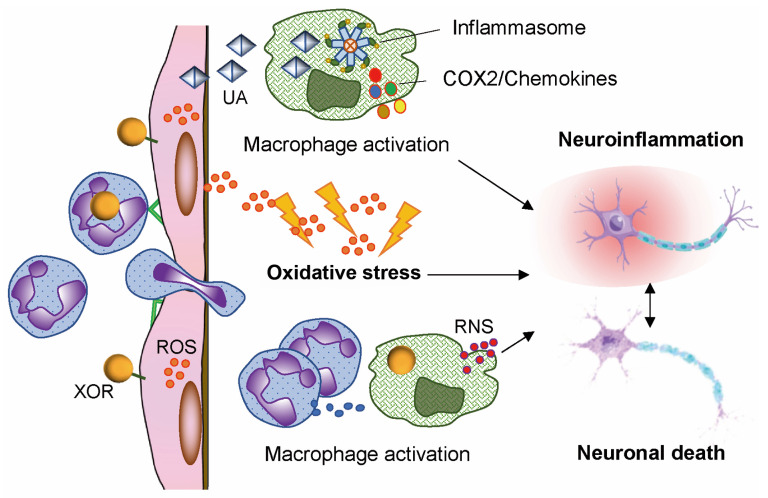
Inflammation, oxidative stress, and neuronal loss elicited by xanthine oxidoreductase (XOR) products. XOR-generated reactive oxygen species (ROS) activate endothelial cells and leukocytes. XOR products can induce tissue damage through the formation of cytotoxic reactive nitrogen species (RNS). Intracellular uric acid (UA) contributes to inflammation and oxidative stress. COX2: Cyclooxygenase-2.

The cytocidal potential of ROS and RNS is functional to the bactericidal capacity of leukocytes during acute inflammation [22] and to the control of the commensal microbiome [11]. However, ROS and RNS are also implicated in the pathological consequences of ischemia and reperfusion injury [23] (Figure 1). Furthermore, they contribute to neuronal death, which can occur as a consequence of chronic neuroinflammation through several pathways, including ferroptosis, an iron-dependent, non-apoptotic, and oxidative form of cell death [24,25] (Figure 2).

A significant body of evidence indicates that oxidative stress plays a crucial role in aging, driven by increased production of ROS and RNS and decreased efficiency of antioxidant scavenger systems. The accumulation of altered molecules, resulting from an excess of free radicals, contributes to chronic inflammatory diseases, characterizing senescence. Additionally, oxidative stress impairs the regenerative capacity of multipotent stem cells. Under pathological conditions, XOR produces harmful free radicals. XOR products, along with increased XOR activity and oxidative stress in aging, contribute to senescence-associated inflammatory responses and related neurodegenerative diseases [26].

## 3. Multiple Sclerosis

Multiple sclerosis (MS) is an autoimmune disease characterized by demyelinating lesions of the CNS induced by chronic inflammation and oxidative stress, with consequent axonal injury and neuronal loss. The disease is linked to the major histocompatibility system, arises in young adults, affects women 2–3 times more than men, and has a chronic course. As the disease progresses, peripheral inflammation and demyelination may develop, giving rise to peripheral neuropathy [3].

Experimental autoimmune encephalomyelitis (EAE) in rodents is a recognized model of MS. After inducing EAE in mice, the effects of SB203580, an inhibitor of the p38 MAP kinase-SGK1 signalling pathway, and of the antioxidant tempol were studied. Both treatments were effective in mitigating disease severity and progression by reducing inflammatory infiltrates and demyelination in the CNS, as well as IL-17 expression levels in the spinal cord and malondialdehyde formation. These findings suggest a pathogenic role of oxidative stress in the development and progression of EAE, and hence MS [27].

In addition to inflammation and oxidative stress, excitotoxicity due to overstimulation of glutamate receptors has also been implicated in the pathogenesis of EAE, as it is associated with elevated ROS/RNS production by the mitochondrial respiratory chain and the induction of lipid peroxidation. In rats with EAE, treatment with glutamate receptor antagonists mitigated the severity and duration of neurological deficits. Furthermore, such treatment significantly reduced the level of malondialdehyde and prevented the depletion of non-protein-SH groups, while increasing the levels of protein–SH groups, as well as the expression and activity of superoxide dismutase 1 [28].

The development of EAE is associated with an increase in malondialdehyde level, XO activity, and mRNA expression of inducible NO synthase, and a decrease in glutathione (GSH) level in the spinal cord of Dark Agouti rats. Female rats were more susceptible to the disease and developed it more quickly than male rats, who had a more severe neurological deficit that is significantly related to oxidative stress profiles in the spinal cord of male but not female rats. These sex-related differences could be, at least in part, due to males having (a) a higher level of ROS-producing systems, such as XOR activity and inducible NO synthase expression, and (b) a lower level of antioxidant systems, such as superoxide dismutase activity and GSH [29].

In a model of EAE induced in female Dark Agouti rats, the inflammatory and redox status of pituitary and adrenal gland tissues was studied. Both the proinflammatory cytokine tumour necrosis factor and inducible NO synthase were upregulated, and ROS/RNS, such as superoxide anion and NO, accumulated in the pituitary. A significant increase in superoxide anions and malondialdehyde occurred in the pituitary and adrenal glands, where GSH content and catalase activity decreased [30].

To test the hypothesis of a pathogenic role of oxidative stress in MS, 42 Iranian patients with MS were enrolled in a clinical trial and randomly assigned to placebo or antioxidant treatment with N-acetylcysteine 600 mg twice a day. A significant decrease in the serum malondialdehyde level and a significant improvement in the anxiety questionnaire score were observed after 8 weeks in the treated group compared to the control group, thus confirming the initial hypothesis [31].

An inverse correlation was observed between UA levels and disability in 94 MS patients (77% women), assessed by the Expanded Disability Status Scale (EDSS) score. UA levels were significantly lower during a relapse than during a remission period and inversely correlated with EDSS scores when measured outside of a relapse, suggesting that serum UA may serve as a marker of disease activity in MS [32].

A meta-analysis of 12 studies involving 1037 MS patients and 556 controls demonstrated that serum UA levels were lower in MS patients than in healthy controls. UA levels were decreased in MS patients with clinical activity or relapse compared with MS patients with clinical inactivity or remission, although they were not correlated with higher (or lower) EDSS or magnetic resonance imaging activity [33].

In a retrospective longitudinal study of 141 Italian subjects with relapsing-remitting MS, the uricemia level was assessed at the baseline visit and after a 2-year follow-up in relation to clinical relapses, disability progression, and cognitive impairment. Analysis of variance showed a progressive reduction in serum UA levels, suggesting a progressive decrease in antioxidant reserves in relation to the risk of relapse, progression of disability, and impairment of cognitive function [34].

The immune-inflammatory response responsible for neurological lesions in EAE is associated with and favoured by oxidative stress. Probably, a similar process occurs in the pathogenesis of MS, and the antioxidant activity of UA could have a protective role against oxidative stress. On the other hand, the production of ROS/RNS by XOR activity could aggravate the patient’s situation in the case of acidosis or under low oxygenation conditions.

## 4. Alzheimer’s Disease

According to GBD 2016, the number of individuals who lived with dementia doubled compared to 1990, mainly due to increases in population ageing and growth. Among the affected people, two out of three were women. Dementia is the fifth leading cause of death worldwide, and Alzheimer’s disease (AD) is one of the most common causes of dementia in the elderly [35].

AD is characterized by the presence of extracellular amyloid plaques, the main component of which is the pathological peptide amyloid-β (Aβ), together with intracellular neurofibrillary tangles, including hyperphosphorylated Tau protein, which are the molecular hallmark of AD. In addition to age and gender, other risk factors include environmental stress, genetic mutations, and mitochondrial haplotypes. Mitochondrial dysfunction may lead to increased ROS production and promote the formation of AD hallmark molecules, which in turn may contribute to oxidative stress and subsequent lipid peroxidation and protein oxidation [4].

Altered lipid metabolism causing lipid accumulation has been reported in many neurological diseases, such as AD, ALS, HD, MS, and PD [36]. APOE-ε4 is the greatest genetic risk factor for AD by promoting diminished fatty acid catabolism in astrocytes, and consequently the initiation of lipid accumulation. Lipid-laden astrocytes trigger microglial activation that induces neuroinflammation and stimulates neuronal fatty acid oxidation, which generates oxidative stress. Astrocytes are also involved in antioxidant defences based on GSH availability. In vitro studies with human astrocytes showed lower basal GSH secretion by AD cells compared to control cells. Following inflammatory stimulation, GSH secretion also increased in the control cells but not in the AD cells. However, activation of NRF2 transcription by the cruciferous-derived drug sulforaphane was able to reduce amyloid secretion, normalize cytokine release, and increase GSH secretion in AD astrocytes, suggesting a possible therapeutic strategy for AD [37].

Progressive neuronal death in AD occurs as a consequence of sphingolipid overexpression, which increases MAP kinase signalling pathways, while inhibiting the phosphatidylinositol 3-kinase/Akt pathway. These alterations promote depolarization and permeabilization of mitochondria that lead to increased ROS production, starting the apoptosis process with cytochrome c release, Bcl-2 depletion, and caspase-3 activation [38].

UA protected mouse microglia BV2 cells from Aβ toxicity by activating transcription factor EB, which controls autophagosome formation and lysosome function, thereby upregulating the expressions of autophagy-related proteins and facilitating Aβ degradation. UA also activated microglia and upregulated autophagy-related proteins in the hippocampus of Aβ microinjection and APP23/PS45 double transgenic mice models of AD. Furthermore, UA treatment improved cognitive impairment in AD model mice, as demonstrated through various behavioural tests [39].

Following the thesis of a recent publication, AD could be a maladaptation to an evolutionary-based survival pathway based on fructose metabolism and its metabolite UA, which allowed our ancestors to overcome periods of low food availability. A similar metabolic pathway is used by animals before hibernating to promote the reduction of expensive ATP production and the accumulation of fat, which is essential for surviving the winter. The level of intracellular UA is increased by excessive ingestion of fructose or fructose production via the polyol pathway as well as by foods that generate UA. These types of diet induce alterations typical of metabolic syndrome, such as hypertension, accumulation of fat and glycogen in the liver, and insulin resistance, consequently facilitating the availability of glucose for the brain. However, mechanisms that can be advantageous in emergency situations can become injurious if prolonged over time because persistent reduction in ATP production can lead to neuronal atrophy and the onset of cognitive decline [40].

One study, using a database representative of the general UK population, shows that gout is inversely associated with the risk of developing AD. This supports the hypothesis of a neuroprotective role of UA, probably due to its antioxidant properties [41].

Reduced antioxidant defence contributes to lipid peroxidation and protein oxidation, which are associated with neurodegenerative diseases and may be responsible for their initiation and development. Interestingly, postmenopausal reduction in oestrogen levels is associated with increased oxidative exposure and risk of mitochondrial dysfunction that may exacerbate proinflammatory responses and impair synaptic plasticity. Attempts to improve the cellular redox state in AD patients through the administration of antioxidant therapies have not provided encouraging results in the experimental models, possibly due to the treatment beginning at a too advanced stage of the disease [42].

The pathogenesis of AD is complex and includes genetic factors, metabolic disorders, vascular dysfunction, metal exposure, subclinical inflammation, oxidative stress, and excitotoxicity [43]. It is therefore not surprising that the attempt to correct a single factor with antioxidant treatments did not yield positive results.

## 5. Amyotrophic Lateral Sclerosis

Amyotrophic lateral sclerosis (ALS) is a progressive neurodegenerative disorder that affects motor neurons in the brain and spinal cord, thus causing muscle weakness, twitching, and atrophy. Sometimes, ALS depends on a genetic alteration, but most cases are sporadic due to environmental pollutants, such as exposure to lead. ALS has a higher prevalence in men than in women, with a ratio of 1.6:1.

A clinical study was conducted on 251 US patients to determine whether blood UA levels predict ALS outcome. The main outcome measure was survival time. A dose-dependent survival benefit was observed with higher baseline UA levels. Furthermore, the risk of death decreased by 39% for every mg/dL increase in serum urate during the 5 years of follow-up. Both associations between serum UA levels and prolonged survival in ALS were observed in men, but not in women [44]. In a similar way, the clinical outcome was evaluated in 34 Japanese patients aged ≥70 years with ALS. Several biochemical parameters (serum levels of triglycerides, cholesterol, LDL/HDL ratio, glucose, and UA) were investigated. The only observed correlation with survival was the serum UA level [45].

A longitudinal cohort study including 329 Chinese patients (207 men and 122 women) was conducted to explore the association between uricemic levels and survival in ALS. A survival advantage was found in men, but not in women, with higher uricemia, while lower UA levels were associated with shorter survival [46].

A cohort of 942 ALS patients from several Western countries who participated in the phase III EMPOWER clinical trial testing dexpramipexole was used to determine whether the serum UA level is predictive of ALS progression. At the end of the one-year follow-up, patients with uricemia levels at (or above) the population median of 5.1 mg/dL had significantly better outcomes than those with lower values [47].

A meta-analysis including 8 case-control studies and 3 cohort studies was conducted to evaluate the association between ALS and uricemia levels. ALS patients (1168 subjects) had statistically lower levels of UA than 1391 healthy controls. Furthermore, higher serum UA levels were statistically associated with decreased all-cause mortality among 3190 ALS patients [48].

In a large cohort including 841 Italian ALS patients (58.5% men) with extensive cognitive and behavioural assessment performed at diagnosis, higher serum UA levels were associated with higher scores on cognitive–behavioural tests. These findings suggest that a higher uricemia could prevent or delay the deterioration of cognitive function and could be a protective factor for the cognitive–behavioural component of ALS [49].

## 6. Huntington’s Disease

Huntington’s disease (HD) is an autosomal dominant neurodegenerative disease that begins at an average age of 40 years and is fatal 15–20 years later. It affects the basal ganglia by selective degradation of GABAergic medium spiny neurons in the striatum, causing both motor and mental disorders. In women, the symptoms are more severe and the progression more rapid than in men.

The relationship between baseline UA levels and the change in the level of functional decline was analysed for 30 months in 347 HD subjects from the CARE-HD clinical study. The deterioration in total functional capacity over time was inversely related to baseline serum UA levels, suggesting that serum UA levels may be a predictor of the rate of HD progression [50].

The post-mortem frontal lobe and striatum of 14 HD patients were biochemically profiled using an LC-LTQ mass spectrometer and compared to 14 controls. Two spectral features from the frontal lobe and two striatum biomarkers had 83% and 91.8% predictive accuracy for HD, respectively. A number of metabolic disturbances were evidenced, including UA levels, which were altered in both the frontal lobe and striatum, highlighting the utility of high-resolution metabolomics for the study of HD [51].

UA levels were lower in post-mortem prefrontal cortical samples from 10 HD patients compared to 10 pre-symptomatic HD subjects. Plasma and salivary samples were obtained from 107 and 178 subjects, respectively, including HD patients, pre-symptomatic HD subjects, and control subjects (54% women). Both plasma and salivary UA levels were (i) significantly lower in pre-symptomatic HD women and HD men compared to control subjects, and (ii) significantly correlated with the loss of total functional capacity and motor ability in HD men. Uricemia levels were significantly correlated with the loss of motor ability in HD women [52].

## 7. Parkinson’s Disease

Over the last 25 years, the number of Parkinson’s disease (PD) patients has more than doubled due to two main factors: longer lifespans, resulting in a longer period of aging, and improved treatments that extend the duration of the disease. PD is characterized by rest tremor, bradykinesia, stiffness of limbs and torso, and postural instability caused by progressive neurodegeneration of dopaminergic neurons. Men are affected 1.4 times more than women. In addition to age and gender, other risk factors are industrial chemicals and pollutants, such as pesticides, solvents, and metals [53].

Aggregation of misfolded α-synuclein is the pathological hallmark of PD and prospectively also forms the basis for its diagnosis by a recently proposed seed amplification assay [54].

In the pathogenesis of PD, activated microglia promote neuronal oxidative stress and lipid peroxidation by producing ROS, while reactive astrocytes release inflammatory cytokines, which promote iron accumulation in neurons, thus inducing ferroptosis [55].

Autophagy is a highly conserved cellular process that profoundly impacts the efficacy of genotoxic chemotherapeutic drugs, such as gemcitabine, that induce autophagy in HeLa cells through the action of UA, which induces phosphorylation of TGF-β-activated kinase 1 and activates the AMP-activated protein kinase (AMPK)/ULK1 pathway leading to autophagy. Gemcitabine-induced autophagy was effectively blocked by the use of the XOR inhibitor allopurinol or by knockdown of the *XOR* gene [56].

Treatment with UA resulted in autophagy activity enhancement in PC12 cells, a well-established cellular model for PD. UA promotes α-synuclein degradation in cells overexpressing it and in SNCAA53T transgenic mice, in which UA increases autophagy by inhibiting the mTor/ULK1 signalling pathway [57]. Other cellular and animal experiments did not confirm the induction of autophagolysosome by UA; however, they demonstrated that increased UA was able to decrease neuron-to-neuron propagation of α-synuclein in vitro and to increase the survival of mice by reducing the expression of phosphorylated α-synuclein in the substantia nigra [58].

Glutathione depletion has been reported in the brains of both PD patients and animal models. Low GSH concentrations in all tissues, including the brain, were observed in mice after deletion of the rate-limiting GSH synthesis gene glutamate-cysteine ligase modifier subunit. In this mouse strain, the herbicide paraquat caused oxidative stress and selective degeneration of nigral dopaminergic neurons, resulting in a PD-like neurobehavioral syndrome. This study suggests that PD results from persistent oxidative stress in the presence of a deficiency in antioxidant systems [59].

The role of oxidative stress in the pathogenesis of PD has been further elucidated by studies on PD-causative mutations. However, none of the antioxidants tested so far in clinical trials, such as creatine, vitamin E, coenzyme Q10, pioglitazone, melatonin, and desferrioxamine, significantly improved PD patient outcomes [60].

Cultured nigral dopaminergic neurons were protected by urate both directly due to its antioxidant effect and indirectly when cultured in the presence of astrocytes stimulated by urate to produce GSH. In vitro and animal experiments suggest that urate protects neurons by activating the NRF2 antioxidant pathway [2].

Different prospective epidemiological and cohort clinical studies have shown that hyperuricemia is associated with a lower risk of developing PD, as well as with a slower progression of the disease.

A cross-sectional case-control study investigated the relevance of uricemia level or caffeine intake, as both UA and caffeine are able to stimulate the nervous system, in US patients with idiopathic PD (236 men, 133 women) compared to the controls. Caffeine consumption was significantly lower in male PD patients, but not in females, compared to the controls. A higher uricemia level was associated with a lower risk of developing PD in both men and women [61].

The level of uricemia was followed for two years in a cohort of patients with a genetic form of PD harbouring mutations in the glucocerebrosidase gene. The results suggest that a low uricemic level might be a progression biomarker in this type of PD, consistent with reports regarding other genetic forms of PD [62]. Somewhat contrasting results were obtained in a longitudinal study with a 5-year follow-up of a cohort of 423 US PD patients compared to 196 healthy controls. Parkinsonian motor deficits were evaluated using dopamine transporter ligand brain scans and the Unified Parkinson’s Disease Rating Scale (UPDRS). The results demonstrate that the level of uricemia does not vary significantly, thus not tracking the increasing severity of PD symptoms [63].

In a clinical study with Chinese subjects, the uricemia levels of 88 PD patients were significantly lower than those of 68 healthy controls. Furthermore, the level of uricemia was significantly negatively correlated with the disease course and nonmotor symptoms, such as dysphagia, anxiety, depression, apathy, and cognitive dysfunction, while it was significantly positively correlated with the grey matter volume in the whole brain [64]. However, a systematic review of the literature was conducted to evaluate the relationship between uricemia level and nonmotor symptoms in 1104 PD patients, without reaching a definitive result [65].

Uricemia levels and clinical imaging of dopaminergic degeneration in 423 de novo PD patients and in subjects with prodromal PD symptoms (39 subjects with REM sleep behaviour disorder and 26 hyposmia patients) were compared to those of 196 healthy controls. Data were extracted from the 5-year longitudinal study of the Parkinson’s Progression Markers Initiative. In the prodromal PD groups, with evidence of dopaminergic degeneration, longitudinal uricemia levels were not statistically different from those of the control group, but were statistically higher than those of the established PD cohort, indicating that the decrease in the levels of uricemia occurs with the transition from prodromal to clinical PD [66].

Some genetic forms of PD are associated with a mutation in the gene for α-synuclein, a neuronal protein regulating synaptic vesicle trafficking that can accumulate in Lewy bodies with an amyloid-like cross-sheet structure [67,68]. In a *Caenorhabditis elegans* model of PD, the *xdh-1* mutation, which reduces XOR activity and ROS formation in dopaminergic neurons, is protective against neurodegeneration, attributed to α-synuclein-induced oxidative stress. Notably, higher levels of UA are associated with lower XOR activity and ROS production in this model [69]. Dopaminergic neurodegeneration has also been observed in *C. elegans* after exposure to soil bacteria that induce oxidative stress and mitochondrial dysfunction. Under these conditions, a calcium-dependent conversion of XDH to XO occurs, which in turn enhances neurodegeneration, possibly through ROS production [70].

Compared to 13 Japanese control patients with different neurological diseases, serum XO activity was markedly upregulated in 41 PD patients and also significantly correlated with disease severity. No correlation was found between serum XO activity and UA levels, which were statistically lower in PD patients compared to controls [71].

A records-based longitudinal study evaluated the US population older than 66 years, with a 5-year follow-up, comparing 42,885 patients with AD, PD, and/or ALS to 207,764 control subjects free of neurodegenerative diseases with respect to pharmacological treatment with allopurinol. XOR inhibition was associated with a significant but modestly reduced risk of all the three neurodegenerative diseases. It is unclear whether this finding was somehow due to a previous higher level of UA or to a reduction in XOR-related oxidative stress [72].

## 8. Stroke

**Figure 3:**
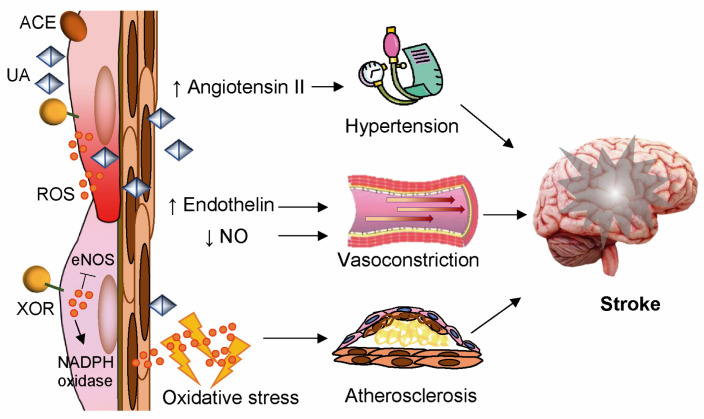
Xanthine oxidoreductase (XOR) products induce vascular alterations favouring stroke. Pro-atherosclerotic stimuli augment the expression and activity of endothelial XOR and its production of reactive oxygen species (ROS), which inactivate endothelial nitric oxide synthase (eNOS) and activate nicotinamide adenine dinucleotide phosphate (NADPH) oxidase. Uric acid (UA) stimulates the expression of endothelin. In addition, an increased level of UA-derived free radicals inside endothelial cells decreases nitric oxide (NO) bioavailability and promotes vasoconstriction. Hyperuricemia also promotes hypertension through the formation of angiotensin II by upregulating the renin–angiotensin system through the angiotensin-converting enzyme (ACE).

Globally, ischemic and haemorrhagic strokes are the second leading cause of death and the third cause of disability-adjusted life-years, with a prevalence of ischemic over haemorrhagic modality. In 2019, the five leading risk factors for stroke were high systolic blood pressure, high body mass index, high fasting plasma glucose, ambient particulate matter pollution, and smoking [73]. These risk factors largely coincide with those that increase endothelium-associated XOR activity and the production of ROS and UA-associated free radicals. ROS inactivate endothelial nitric oxide synthase (eNOS) and activate NADPH oxidase [74]. UA-derived free radicals inside endothelial cells decrease NO bioavailability and promote vasoconstriction. In addition, hyperuricemia stimulates the expression of endothelin and promotes hypertension through the formation of angiotensin II by upregulating the renin–angiotensin system [75]. Vascular oxidative stress and the resulting inflammatory responses promote the formation of atheromatous plaques, which can be the site of thrombotic obstruction, which causes ischemia, and weakening of the arterial wall, responsible for haemorrhage [76] (Figure 3).

Data from the National Health and Nutrition Examination Survey from 1999 to 2018 were used to study the relationship between oxidative balance scores and stroke prevalence in 25,258 Chinese participants, aged 20 to 85 years, including 673 subjects with stroke. After adjusting for all confounding factors, diet-related, but not lifestyle-related, oxidative balance scores were inversely associated with stroke prevalence in a dose–response relationship, likely because they indirectly reflect the level of systemic oxidative stress and chronic inflammation. These findings suggest the possibility of preventing stroke by adjusting the daily levels of antioxidants in the diet [77].

In a prospective, single-centre study, 328 Chinese patients with acute ischemic stroke were compared to 107 age- and sex-matched healthy controls to identify the role of serum XO protein levels in stroke. Serum XO levels were significantly higher in patients than in the control group and higher in patients with progressive stroke or poor prognosis than in patients with stable stroke or good prognosis. Furthermore, serum XO concentration at admission was found to be an independent risk factor for the onset of acute ischemic stroke, as well as for its progression and prognosis [78].

Five machine learning models were established to use the serum XO concentration level and clinical data to predict the onset, progression, and prognosis of acute ischemic stroke in 328 Chinese patients compared to 107 healthy controls. These machine learning models could effectively predict the onset and prognosis of acute ischemic stroke, but they were not effective in predicting its progression. The logistic regression learning model has the best predictive performance, showing the highest value of the area under the receiver operating characteristic curve [79].

A drug-target Mendelian randomization analysis was performed using UK Biobank data to investigate the effects of reducing UA levels by XDH inhibition on the risk of ischemic diseases. Furthermore, the population was stratified by UA and lipid levels to assess the outcomes. The risk of cerebral infarction was significantly reduced in subgroups with lower low-density lipoprotein–cholesterol and lower serum UA levels, suggesting the potential benefits of XO inhibition depending on the lipid profile [80].

Various cohort studies and meta-analyses suggest that hyperuricemia may increase the risks of both stroke incidence and mortality, although contrasting results have been reported and a J-shaped relationship between the level of uricemia and the risk of stroke has been also suggested, but only for men [81]. Allopurinol use is significantly associated with a 9% lower hazard ratio for ischemic stroke, after adjusting for age, gender, race, and cardio-protective medications, in a 5% random sample of USA Medicare beneficiaries from 2006 to 2012 [82].

UA provides short-term neuroprotection as an antioxidant; however, hyperuricemia presents long-term risks by promoting hypertension and metabolic disorders that promote cardiorenal and metabolic diseases [83].

## 9. Inosine Treatments

As reported above, several epidemiological studies have shown the positive association of uricemia levels and of appropriate caffeine intake with low incidence of neurodegenerative diseases such as AD, ALS, and PD. The neuroprotective mechanism of caffeine involves adenosine A2A receptor antagonism [84]. In addition, purine derivatives can promote neuronal cysteine uptake and mediate glutathione synthesis for antioxidant neuroprotection [85].

Both experimental and clinical data show an inverse association between UA levels and some neurological diseases, suggesting a protective role of urate against neuronal death induced by oxidative stress. To validate this hypothesis, clinical trials have been formulated to increase the serum urate level and test its feasibility, safety, and therapeutic efficacy in PD and other CNS diseases [86].

The Safety of Urate Elevation in Parkinson’s Disease (SURE-PD) trial demonstrated that inosine supplementation leads to a dose-dependent increase in serum and cerebrospinal fluid urate levels, and it is clinically safe in patients with PD [87]. In the context of the SURE-PD study, a different response to treatment was related to gender. Inosine administration induced a higher elevation of uricemia in women than in men, resulting in a significantly greater cerebrospinal fluid urate only among women. In women, slower rates of clinical decline, as measured by the UPDRS, were associated with greater increases in serum urate and in plasma antioxidant capacity [88]. However, the subsequent Study of Urate Elevation in PD, Phase 3 (SURE-PD3) clinical trial, involving 298 patients with early PD, showed no significant differences in rates of clinical progression or secondary efficacy outcomes among participants in the inosine group and those in the placebo group [89].

A pilot study was conducted on 25 participants with ALS to test inosine treatment for 12 weeks. Inosine appeared to be safe, well tolerated, and capable of increasing serum urate levels in ALS patients and led to increased plasma antioxidant capacity related to changes in serum urate level [90]. However, in the following Safety of Urate Elevation in Amyotrophic Lateral Sclerosis (SURE-ALS2) trial, a functional benefit was not demonstrated [91].

Attempts to increase uricemia levels by inosine treatment gave encouraging results in a small clinical trial with 11 MS patients [92]. Another randomized, double-blind trial included 16 patients with relapsing–remitting MS, with 1-year follow-up. The increased uricemia level obtained through the inosine treatment correlated with a significant decrease in the number of gadolinium-enhanced lesions and improved disability assessed using the Kurtzke Expanded Disability Status Scale, and also with kidney stone formation in 25% of the subjects [93].

The Efficacy Study of Combined Treatment with Uric Acid and rtPA in Acute Ischemic Stroke (URICO-ICTUS) trial included 206 women and 205 men. Excellent outcomes at 90 days were seen in 46% of women treated with UA compared to 29% of women in the placebo group, but no differences were observed in men when comparing the results of treated patients (36%) with those of the placebo group (34%) [94].

The role of UA as an antioxidant in the brain was discussed and denied in a recent review, because the level of UA in the cerebrospinal fluid is low. In addition, inosine treatments increased uricemia levels, but induced only a modest increment in UA levels in the cerebrospinal fluid and did not significantly modify its antioxidant capacity [95]. These findings may explain why inosine treatments did not provide the expected benefits in some patients affected by neurodegenerative diseases.

However, when combined with XOR inhibition, inosine treatment was able to promote the purine salvage pathway, thereby increasing the level of ATP, which was compromised by mitochondrial dysfunction [96].

A US population-based case-control study examined 42,885 patients with neurodegenerative diseases and 334,387 controls aged 66 to 90 years (50% women) to identify which medications used for two years were associated with a lower risk of ALS, AD, and PD. The controls were then followed for the next five years for the development of neurodegenerative diseases. The most protective drug for neurodegenerative diseases has been found to be allopurinol used for gout [72]. This finding raises the question of whether hyperuricemia protects against neurodegenerative diseases or if hyperuricemia treatment with allopurinol is responsible for the protection. A possible answer is provided in the following paragraph.

To understand the role of XOR, UA, and ROS in neurodegenerative diseases, human brain tissue was examined. UA levels were found to be low and XOR was not detected, while hypoxanthine phosphoribosyltransferase was found to be abundant. These results suggest that ROS production by XOR in the brain tissue is irrelevant. To study the role of the purine salvage pathway in brain energy metabolism, XOR and subsequently the XOR inhibitor febuxostat were added to a culture of neurons derived from human-induced pluripotent stem cells. XOR inhibition prevented the loss of the adenylate pool and allowed ATP production primarily through the purine salvage pathway. Since neurons require a lot of energy in the form of ATP, it appears that the action of febuxostat was not focused on inhibiting ROS production but on enabling the action of hypoxanthine phosphoribosyltransferase and that de novo purine biosynthesis is less important than purine salvage activity in neuronal energy production [97].

Encouraging results were obtained in a clinical trial including 26 Japanese PD patients treated for 8 weeks with inosine plus febuxostat. The treatment was relatively safe and effective in improving symptoms assessed through the UPDRS revised by the Movement Disorder Society [98].

## 10. Conclusions

All neurological diseases considered in this review have the following aspects in common: (i) oxidative stress is part of the pathogenetic mechanisms at their origin, and (ii) treatment with inosine has been explored because, except for stroke, the level of uricemia is inversely proportional to their onset and progression. This evidence prompted us to consider the role of XOR in these pathologies.

Human XOR is a highly evolutionarily conserved and particularly versatile enzyme, as its expression can be modulated both at the transcriptional and post-transcriptional levels. It also has various functions because of its various enzymatic activities, specifically XDH, XO, NADH reductase, and nitrate reductase [22]. Furthermore, the low substrate specificity of XOR allows for the metabolism of other different substances, both endogenous and exogenous [99]. In addition, by generating irreversible products, XOR limits the possibility of recycling purines to build new nucleic acids. Finally, XOR generates both oxidant and antioxidant products, and its activity can be blocked by inhibitors approved as drugs. Therefore, it is not surprising that XOR activity and its products have been implicated in the pathogenesis of neurological diseases, as well as proposed as their remedies.

**Figure 4:**
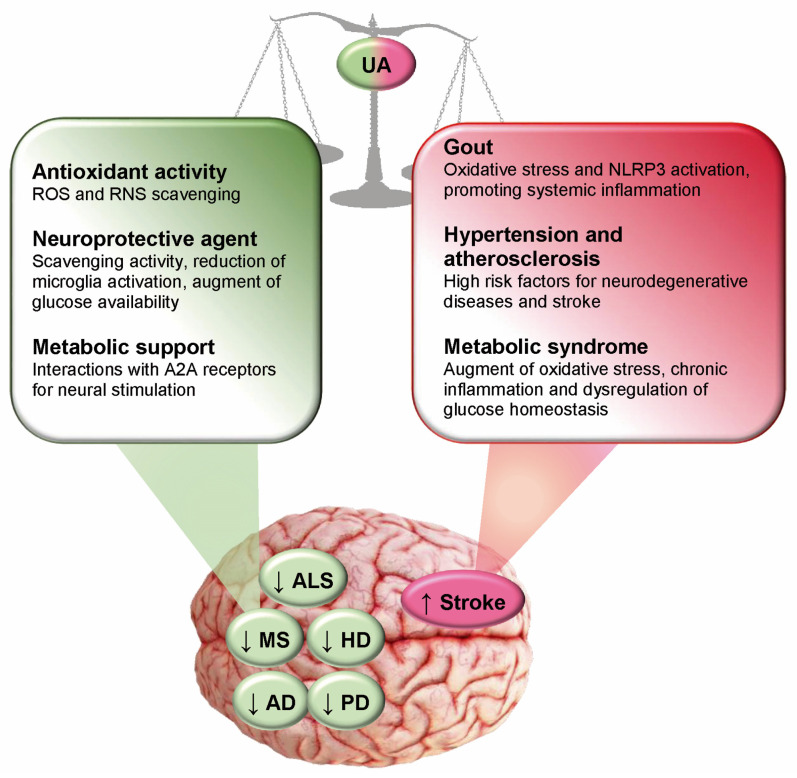
Effects of uric acid on neurodegenerative diseases and stroke. Uric acid (UA) has protective effects on neurodegenerative diseases, exerting antioxidant and neuroprotective activities and providing metabolic support to neurons. However, hyperuricemia is protective against ischemic damage (stroke) only during the initial phase of UA increase, while a long-term increase in UA can trigger oxidative stress and an inflammatory response, aggravating brain tissue damage. Furthermore, it should be noted that since a high level of UA is a risk factor for cardiovascular diseases and metabolic syndrome, it is consequently also a risk factor for stroke. AD: Alzheimer’s Disease; ALS: Amyotrophic Lateral Sclerosis; HD: Huntington’s Disease; MS: Multiple Sclerosis; NLRP: Nucleotide-binding oligomerization domain, Leucine rich Repeat and Pyrin domain containing; PD: Parkinson’s Disease.

The inverse correlation observed between the level of uricemia and the presence of some neurological diseases suggests a neuroprotective role for UA (Figure 4). The most obvious explanation would be based on the antioxidant properties of UA, although the possibility that it can reach sufficient levels in the cerebrospinal fluid is disputed. The prevalence of AD in females after menopause could be explained by the further decrease in antioxidant capacity due to the decline in oestrogen activity. However, the ability of purines to stimulate the central nervous system through interaction with the A2A receptors for adenosine should also be considered.

The neuroprotective action of UA has been ascribed to its ability to (i) promote the NRF2 nuclear translocation and, consequently, the expression of NRF2-targeted antioxidant genes, (ii) reduce neuroinflammation due to cytokine gene expression by inhibiting the activation of microglia, (iii) protect the blood–brain barrier by lowering the level of vascular endothelial growth factor A, which has a permeabilising action, (iv) increase the autophagy of α-synuclein intraneuronal aggregates by activating the AMP-activated protein kinase, and (v) protect against the deposition of extracellular misfolded proteins, such as Aβ accumulation and the hyperphosphorylated microtubule-associated tau protein [9].

A beneficial effect was also produced by UA treatment administered to male Wistar rats subjected to transient ischemic stroke. The UA-treated animals had a smaller infarct, less oedema, reduced apoptosis, and improved neurofunctional status [100].

Although cellular and animal experiments are useful in shedding light on the molecular mechanisms of neurodegenerative diseases, not all findings can be translated into clinical practice in humans due to significant differences between the biology and pathophysiology of rodents and humans. Furthermore, gender differences must be taken into account, such as the influence of oestrogens on antioxidant defences, which may be relevant in the distribution of the disease as well as in the response to therapy [46,52,88,94,101]. Additionally, potential interferences due to ethnicity should be noted when population studies concern subjects from a single nation.

However, UA protection of brain tissue appears to be effective only during the initial stage of elevated UA levels, whereas long-term UA elevation may trigger an inflammatory response and be responsible for aggravating brain tissue damage [102].

Attempts to increase uricemia have so far had poor therapeutic results. Inosine treatments may benefit from the concomitant administration of XOR inhibitors to reduce the risk of fuelling oxidative stress and to allow purine salvage to facilitate ATP synthesis. It is essential to test inosine treatment at an early stage of the pathology, with the aim of reducing the spread of neuronal damage.

The prospects opened up by the results of the inosine treatment and febuxostat suggest that this path should be further explored to verify whether the inhibition of XOR, and the consequent enhancement of the purine salvage pathway, is effective in energy production and neuronal protection. Treatment should possibly be started at the prodromal stage of the neurodegenerative disease, before the damage is widely spread.

These considerations paint a rather complicated picture, where it is not easy to reach the right balance. In fact, on the one hand, by promoting uricemia increase, the risk factors for atherosclerosis are simultaneously promoted, and on the other hand, by inhibiting XOR, the positive aspects linked to the production of ROS and RNS are negated.

Among the new therapeutic approaches to targeting neurodegenerative disorders, a new line of interventions based on the administration of neurotrophic genes should be mentioned. Nerve growth factor genes can be effective in promoting neuronal growth and can be administered to the affected region using viral or non-viral vectors. Gene therapy promises to ensure the survival of neurons as long as it is started at an early stage of the disease, before the damage is too extensive [103].

## Abbreviations

The following abbreviations are used in this manuscript:

Aβ	Amyloid-β
ACE	Angiotensin-Converting Enzyme
AD	Alzheimer’s Disease
ALS	Amyotrophic Lateral Sclerosis
CNS	Central Nervous System
COX2	Cyclooxygenase-2
EAE	Experimental Autoimmune Encephalomyelitis
EDSS	Expanded Disability Status Scale
ENOS	Endothelial Nitric Oxide Synthase
GBD	Global Burden of Diseases, Injuries, and Risk Factors Study
GSH	Reduced Glutathione
HD	Huntington’s Disease
MAP	Mitogen-Activated Protein
MS	Multiple Sclerosis
NAD	Nicotinamide Adenine Dinucleotide
NADH	Nicotinamide Adenine Dinucleotide Reduced
NADP	Nicotinamide Adenine Dinucleotide Phosphate
NADPH	Nicotinamide Adenine Dinucleotide Phosphate Reduced
NO	Nitric Oxide
NRF2	Nuclear Factor Erythroid 2-Related Factor 2
PD	Parkinson’s Disease
RNS	Reactive Nitrogen Species
ROS	Reactive Oxygen Species
UA	Uric Acid
UPDRS	Unified Parkinson’s Disease Rating Scale
XDH	Xanthine Dehydrogenase
XO	Xanthine Oxidase
XOR	Xanthine Oxidoreductase
